# Supplementary material for: The Allelopathic Potential of Rosa blanda Aiton on Selected Wild-Growing Native and Cultivated Plants in Europe
Source: Plants (Basel). 2021 Aug 30;10(9):1806. doi: 10.3390/plants10091806 (PMC8471273; doi:10.3390/plants10091806)
Supplement: Supplementary file 1 [file plants-10-01806-s001.zip › plants-1350932-supplementary.pdf]

plants

Table S1. F and p values for selected germination and growth parameters of the tested Red fescue (*Festuca rubra* L.) and Red radish (*Raphanus sativus* L. var. *radicula* Pers. cv. Rowa) seedlings treated with aqueous extracts from the organs of *Rosa blanda* Aiton.

**Extract type**	**GP in 7 day..**			
	Red fescue		Red radish	
	F	*p	F	*p
**Root**	2.32	0.000	4.09	0.035
**Stalk**	5.23	0.000	2.57	0.11 ns
**Leaf**	4.61	0.000	6.17	0.01
**Flower**	6.82	0.000	3.70	0.000

**Extract type**	**RI**			
	Red fescue		Red radish	
	F	*p	F	*p
**Root**	5.23	0.048	5.71	0.041
**Stalk**	8.41	0.003	1.90	0.029
**Leaf**	5.81	0.000	3.85	0.006
**Flower**	3.09	0.000	6.76	0.000

**Extract type**	**EC**			
	Red fescue		Red radish	
	F	*p	F	*p
**Root**	10.33	0.000	3.23	0.033
**Stalk**	4.99	0.006	2.19	0.000
**Leaf**	8.83	0.000	3.38	0.029
**Flower**	12.5	0.000	7.49	0.001

**Extract type**	Length of whole seedling			
	Red fescue		Red radish	
	F	*p	F	*p
**Root**	7.73	0.000	6.98	0.000
**Stalk**	5.27	0.000	9.37	0.000
**Leaf**	4.18	0.000	8.27	0.000
**Flower**	3.67	0.000	6.54	0.000

GP – germination percentage in 7 day of germination, RI – allelopathic seeds response, EC – electrolyte leakage, *p – statistically significant $p \leq 0.05$, ns – statistically insignificant
